# Histogram of Gradient Orientations of Signal Plots Applied to P300 Detection

**DOI:** 10.3389/fncom.2019.00043

**Published:** 2019-07-05

**Authors:** Rodrigo Ramele, Ana Julia Villar, Juan Miguel Santos

**Affiliations:** Computer Engineering Department, Centro de Inteligencia Computacional, Instituto Tecnológico de Buenos Aires (ITBA), Buenos Aires, Argentina

**Keywords:** Electroencephalography, Histogram of Gradient Orientations, brain-computer interfaces, P300, SIFT, amyotrophic lateral sclerosis, naive-bayes near neighbors, waveforms

## Abstract

The analysis of Electroencephalographic (EEG) signals is of ulterior importance to aid in the diagnosis of mental disease and to increase our understanding of the brain. Traditionally, clinical EEG has been analyzed in terms of temporal waveforms, looking at rhythms in spontaneous activity, subjectively identifying troughs and peaks in Event-Related Potentials (ERP), or by studying graphoelements in pathological sleep stages. Additionally, the discipline of Brain Computer Interfaces (BCI) requires new methods to decode patterns from non-invasive EEG signals. This field is developing alternative communication pathways to transmit volitional information from the Central Nervous System. The technology could potentially enhance the quality of life of patients affected by neurodegenerative disorders and other mental illness. This work mimics what electroencephalographers have been doing clinically, visually inspecting, and categorizing phenomena within the EEG by the extraction of features from images of signal plots. These features are constructed based on the calculation of histograms of oriented gradients from pixels around the signal plot. It aims to provide a new objective framework to analyze, characterize and classify EEG signal waveforms. The feasibility of the method is outlined by detecting the P300, an ERP elicited by the oddball paradigm of rare events, and implementing an offline P300-based BCI Speller. The validity of the proposal is shown by offline processing a public dataset of Amyotrophic Lateral Sclerosis (ALS) patients and an own dataset of healthy subjects.

## 1. Introduction

Although recent advances in neuroimagining techniques, particularly radio-nuclear and radiological scanning methods (Schomer and Silva, 2010), have diminished the prospects of the traditional Electroencephalography, the advent and development of digitized devices has impelled for a revamping of this hundred years old technology. Their versatility, ease of use, temporal resolution, ease of development and production, and its proliferation as consumer devices, are pushing EEG to become the de-facto non invasive portable or ambulatory method to access and harness brain information (De Vos and Debener, 2014).

A key contribution to this expansion has been the field of Brain Computer Interfaces (Wolpaw and Wolpaw, 2012) which is the pursuit of the development of a new channel of communication particularly aimed to persons affected by neurodegenerative diseases.

One noteworthy aspect of this novel communication channel is the ability to transmit information from the Central Nervous System (CNS) to a computer device and from there use that information to control a wheelchair (Carlson and del R. Millan, 2013), as input to a speller application (Guger et al., 2009), in a Virtual Reality environment (Lotte et al., 2013) or as aiding tool in a rehabilitation procedure (Jure et al., 2016). The holly grail of BCI is to implement a new complete and alternative pathway to restore lost locomotion (Wolpaw and Wolpaw, 2012).

EEG signals are remarkably complex and have been characterized as a multichannel non-stationary stochastic process. Additionally, they have high variability between different subjects and even between different moments for the same subject, requiring adaptive and co-adaptive calibration and learning procedures (Clerc et al., 2016). Hence, this imposes an outstanding challenge that is necessary to overcome in order to extract information from raw EEG signals.

BCI has gained mainstream public awareness with worldwide challenge competitions like Cybathlon (Riener and Seward, 2014; Novak et al., 2018) and even been broadcasted during the inauguration ceremony of the 2014 Soccer World Cup. New developments have overcome the out-of-the-lab high-bar and they are starting to be used in real world environments (Huggins et al., 2016; Guger et al., 2017). However, they still lack the necessary robustness, and its performance is well behind any other method of human computer interaction, including any kind of detection of residual muscular movement (Clerc et al., 2016).

A few works have explored the idea of exploiting the signal waveform to analyze the EEG signal. In Alvarado-González et al. (2016), an approach based on Slope Horizontal Chain Code is presented, whereas in Yamaguchi et al. (2009) a similar procedure was implemented based on Mathematical Morphological Analysis. The seminal work of Bandt-Pompe Permutation Entropy (Berger et al., 2017) also explores succinctly this idea as a basis to establish the time series ordinal patterns. In the article (Ramele et al., 2016), the authors introduce a method for classification of rhythmic EEG events like Visual Occipital Alpha Waves and Motor Imagery Rolandic Central μ Rhythms using the Histogram of Gradient Orientations of signal plots. Inspired in that work, we propose a novel application of the developed method to classify and describe transient events, particularly the P300 Event Related Potential. The proposed approach is based on the waveform analysis of the shape of the EEG signal. The signal is drawn on a bidimensional image plot, vector gradients of pixels around the plot are obtained, and with them, the histogram of their orientations is calculated. This histogram is a direct representation of the waveform of the signal. The method is built by mimicking what regularly electroencephalographers have been performing for almost a century as it is described in Hartman (2005): visually inspecting raw signal plots.

This paper reports a method to: (1) describe a procedure to capture the shape of a waveform of an ERP component, the P300, using histograms of gradient orientations extracted from images of signal plots, and (2) outline the way in which this procedure can be used to implement an P300-Based BCI Speller application. Its validity is verified by offline processing two datasets, one of data from ALS patients and another one from data of healthy subjects.

This article unfolds as follows: section 2.1 is dedicated to explain the Feature Extraction method based on Histogram of Gradient Orientations of the Signal Plot, section 2.1.1 shows the preprocessing pipeline, section 2.1.2 describes the image generation of the signal plot, section 2.1.3 presents the feature extraction procedure while section 2.1.4 introduces the Speller Matrix Letter Identification procedure. In section 2.2, the experimental protocol is expounded. Section 3 shows the results of applying the proposed technique. In the final section 4 we expose our remarks, conclusions, and future work.

## 2. Materials and Methods

**Figure 1:**
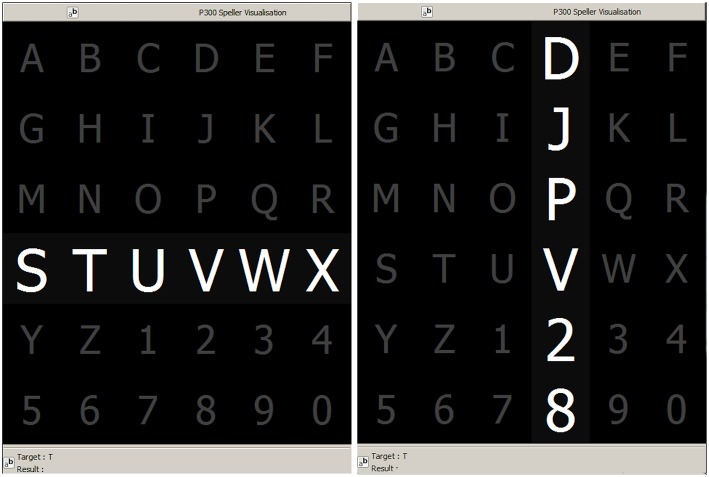
Example of the 6 × 6 Speller Matrix used in the study obtained from the OpenVibe software. Rows and columns flash in random permutations.

The P300 (Farwell and Donchin, 1988; Knuth et al., 2006) is a positive deflection of the EEG signal which occurs around 300 ms after the onset of a rare and deviant stimulus that the subject is expected to attend. It is produced under the oddball paradigm (Wolpaw and Wolpaw, 2012) and it is consistent across different subjects. It has a lower amplitude (±5μ*V*) compared to basal EEG activity, reaching a Signal to Noise Ratio (SNR) of around −15 db estimated based on the amplitude of the P300 response signal divided by the standard deviation of the background EEG activity (Hu et al., 2010). This signal can be used to implement a speller application by means of a Speller Matrix (Farwell and Donchin, 1988). This matrix is composed of 6 rows and 6 columns of numbers and letters. The subject can focus on one character of the matrix. Figure 1 shows an example of the Speller Matrix used in the OpenVibe open source software (Renard et al., 2010), where the flashes of rows and columns provide the deviant stimulus required to elicit this physiological response. Each time a row or a column that contains the desired letter flashes, the corresponding synchronized EEG signal should also contain the P300 signature and by detecting it, the selected letter can be identified.

### 2.1. Feature Extraction From Signal Plots

**Figure 2:**
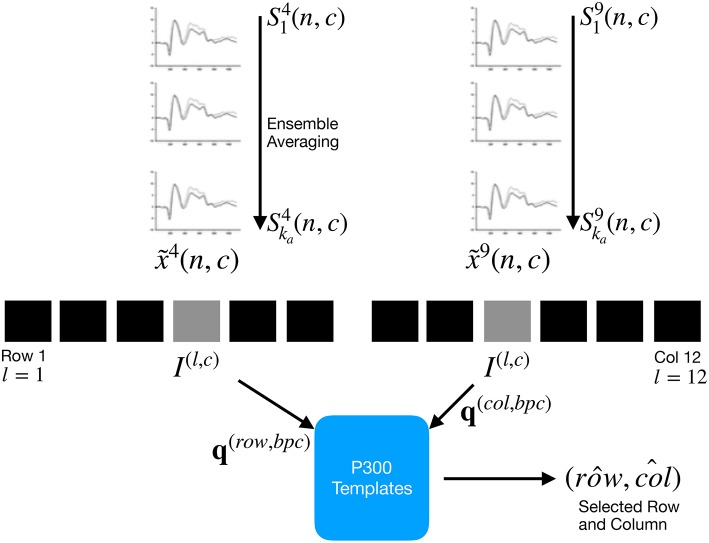
For each column and row, an averaged, standardized and scaled signal ${\tilde{x}}^{l}\left(n,c\right)$ is obtained from the segments $S_{i}^{l}$ corresponding to the *k*_*a*_ intensification sequences with 1 ≤ *i* ≤ *k*_*a*_ and location *l* varying between 1 and 12. From the averaged signal, the image *I*^(*l,c*)^ of the signal plot is generated and each descriptor is computed. By comparing each descriptor against the set of templates, the P300 ERP can be detected, and finally the desired letter from the matrix can be inferred.

In this section, the signal preprocessing, the method for generating images from signal plots, the feature extraction procedure and the Speller Matrix identification are described. Figure 2 shows a scheme of the entire process.

#### 2.1.1. Preprocessing Pipeline

The data obtained by the capturing device is digitalized and a multichannel EEG signal is constructed.

The 6 rows and 6 columns of the Speller Matrix are intensified providing the visual stimulus. The number of a row or column is a location. A sequence of 12 randomly permuted locations *l* conform an intensification sequence. The whole set of 12 intensifications is repeated *k*_*a*_ times.

- **Signal Enhancement**: This stage consists of the enhancement of the SNR of the P300 pattern above the level of basal EEG. The pipeline starts by applying a notch filter to the raw digital signal, a 4th degree 10 Hz lowpass Butterworth filter and finally a decimation with a Finite Impulse Response (FIR) filter of order 30 from the original sampling frequency down to 16 Hz (Krusienski et al., 2006).
- **Artifact Removal**: For every complete sequence of 12 intensifications of 6 rows and 6 columns, a basic artifact elimination procedure is implemented by removing the entire sequence when any signal deviates above/bellow ±70μ*V*.
- **Segmentation**: For each of the 12 intensifications of one intensification sequence, a segment $S_{i}^{l}$ of a window of *t*_*max*_ seconds of the multichannel signal is extracted, starting from the stimulus onset, corresponding to each row/column intensification *l* and to the intensification sequence *i*. As intensifications are permuted in a random order, the segments are rearranged corresponding to row flickering, labeled 1–6, whereas those corresponding to column flickering are labeled 7–12. Two of these segments should contain the P300 ERP signature time-locked to the flashing stimulus, one for the row, and one for the column.
- **Signal Averaging**: The P300 ERP is deeply buried under basal EEG so the standard approach to identify it is by point-to-point averaging the time-locked stacked signal segments. Hence the values which are not related to, and not time-locked to the onset of the stimulus are canceled out (Liang and Bougrain, 2008).

This last step determines the operation of any P300 Speller. In order to obtain an improved signal in terms of its SNR, repetitions of the sequence of row/column intensification are necessary. And, at the same time, as long as more repetitions are needed, the ability to transfer information faster is diminished, so there is a trade-off that must be acutely determined.

The procedure to obtain the point-to-point averaged signal goes as follows:

1. Highlight randomly the rows and columns from the matrix. There is one row and one column that should match the letter selected by the subject.
2. Repeat step 1 *k*_*a*_ times, obtaining the 1 ≤ *l* ≤ 12 segments $S_{1}^{l}\left(n,c\right),\ldots ,S_{k_{a}}^{l}\left(n,c\right)$, of the EEG signal where the variables 1 ≤ *n* ≤ *n*_*max*_ and 1 ≤ *c* ≤ *C* correspond to sample points and channel, respectively. The parameter *C* is the number of available EEG channels whereas *n*_*max*_ = *F*_*s*_ *t*_*max*_ is the segment length and *F*_*s*_ is the sampling frequency. The parameter *k*_*a*_ is the number of repetitions of intensifications and it is an input parameter of the algorithm.
3. Compute the Ensemble Average by
  (1) $$\begin{array}{r} x^{l}\left(n,c\right)=\frac{1}{k_{a}}\overset{k_{a}}{\underset{i=1}{\sum }}S_{i}^{l}\left(n,c\right) \end{array}$$
  for 1 ≤ *n* ≤ *n*_*max*_ and for the channels 1 ≤ *c* ≤ *C*. This provide an averaged signal *x*^*l*^(*n, c*) for the twelve locations 1 ≤ *l* ≤ 12.

#### 2.1.2. Signal Plotting

Averaged signal segments are standardized and scaled for 1 ≤ *n* ≤ *n*_*max*_ and 1 ≤ *c* ≤ *C* by

(2) $${\tilde{x}}^{l}(n,c)=\left\lfloor \gamma \;\frac{\left(x^{l}(n,c)-{\bar{x}}^{l}(c)\right)}{{\hat{\sigma }}^{l}(c)}\right\rfloor$$

where γ > 0 is an input parameter of the algorithm and it is related to the image scale. In addition, *x*^*l*^(*n, c*) is the point-to-point averaged multichannel EEG signal for the sample point *n* and for channel *c*. Lastly,

$${\bar{x}}^{l}\left(c\right)=\frac{1}{n_{max}}\overset{n_{max}}{\underset{n=1}{\sum }}x^{l}\left(n,c\right)$$

and

$${\hat{\sigma }}^{l}(c)={\left\{\frac{1}{n_{max}-1}\sum_{n=1}^{n_{max}}{\left[x^{l}(n,c)-{\bar{x}}^{l}(c)\right]}^{2}\right\}}^{\frac{1}{2}}$$

are the mean and estimated standard deviation of $x^{l}\left(n,c\right),1\leq n\leq n_{max}$, for each channel *c*.

Consequently, a binary image *I*^(*l,c*)^ is constructed according to

(3) $$I^{\left(l,c\right)}(z_{1},z_{2})=\left\{ \begin{array}{l} \text{255 if }z_{1}=\gamma \;n\text{ and ​}z_{2}={\tilde{x}}^{l}(n,c)+z^{l}(c)\\ \text{ 0 otherwise} \end{array}\right.$$

with 255 being white and representing the signal's value location and 0 for black which is the background contrast, conforming a black-and-white plot of the signal. Pixel arguments (*z*_1_, *z*_2_)∈ℕ × ℕ iterate over the width (based on the length of the signal segment) and height (based on the peak-to-peak amplitude) of the newly created image with 1 ≤ *n* ≤ *n*_*max*_ and 1 ≤ *c* ≤ *C*. The value *z*^*l*^(*c*) is the image vertical position where the signal's zero value has to be situated in order to fit the entire signal within the image for each channel c:

(4) $$z^{l}(c)=\left\lfloor \frac{\max_{n}{\tilde{x}}^{l}(n,c)-\min_{n}{\tilde{x}}^{l}(n,c)}{2}\right\rfloor -\left\lfloor \frac{\max_{n}{\tilde{x}}^{l}(n,c)+\min_{n}{\tilde{x}}^{l}(n,c)}{2}\right\rfloor$$

where the minimization and maximization are carried out for *n* varying between 1 ≤ *n* ≤ *n*_*max*_, and ⌊·⌋ denote the rounding to the smaller nearest integer of the number.

In order to complete the plot *I*^(*l, c*)^ from the pixels, the Bresenham (Bresenham, 1965; Ramele et al., 2016) algorithm is used to interpolate straight lines between each pair of consecutive pixels.

#### 2.1.3. Feature Extraction: Histogram of Gradient Orientations

The work of Hubel and Wiesel (1962), on how the visual cortex sense features was the inspiration to the development of an algorithm to identify and decode salient local information from image regions. The Scale Invariant Feature Transform (SIFT) is a Computer Vision method proposed by Lowe (2004) which is composed of two parts, the SIFT Detector and the SIFT Descriptor. The former is the procedure to identify relevant areas of an image whereas the latter is the procedure to describe and characterize a region of an image (i.e. patch) calculating an histogram of the angular orientations of pixel gradients. In order to characterize EEG signal waveforms, this work proposes an alternative to the SIFT Descriptor, the Histogram of Gradient Orientations (HIST) algorithm.

For each generated image *I*^(*l, c*)^, a keypoint **p_*k*_** is placed on a pixel (*x*_*p*_*k*__, *y*_*p*_*k*__) over the image plot and a window around the keypoint is considered: a local image patch. Its size is *X*_*p*_ × *X*_*p*_ pixels and is constructed by dividing the window in 16 blocks of size 3*s* each one, where *s* is the scale of the local patch and it is an input parameter of the algorithm. It is arranged in a 4 × 4 grid and the pixel **p_*k*_** is the patch center, thus *X*_*p*_ = 12*s* pixels.

A local representation of the signal shape within the patch can be described by obtaining the gradient orientations on each of the 16 blocks *B*_*i, j*_ with 0 ≤ *i, j* ≤ 3 and creating a histogram of gradients. In order to calculate the histogram, the interval [0, 360] of possible angles is divided in 8 bins, each one of 45 degrees.

Hence, for each spatial bin 0 ≤ *i, j* ≤ 3, corresponding to the indexes of each block *B*_*i, j*_, the orientations are accumulated in a 3-dimensional histogram *h* through the following equation:

(5) $$h(\theta ,i,j)=3s\sum_{p\in I^{\left(l,c\right)}}w_{\text{ang}}(∠J(p)-\theta )w_{ij}\left(\frac{p-p_{k}}{3s}\right)\left\|J(p)\right\|$$

where **p** is a pixel from the image *I*^(*l, c*)^, θ is the angle bin with θ∈{0, 45, 90, 135, 180, 225, 270, 315}, ‖*J*(**p**)‖ is the euclidean norm of the gradient vector in the pixel **p** and it is computed using finite differences and ∠*J*(**p**) is the angle of the gradient vector.

The contribution of each gradient vector to the histogram calculated by Equation 5 is balanced by a trilinear interpolation. The scalar *w*_ang_(·) and vector *w*_*ij*_(·) functions are linear interpolations used by Lowe (2004) and Vedaldi and Fulkerson (2010) to provide a weighting contribution to the eight adjacent bins in the tridimensional histogram. They are calculated as

(6) $$\begin{array}{r} w_{ij}\left(\text{v}\right)=w\left(v_{x}-x_{i}\right)w\left(v_{y}-y_{j}\right) \end{array}$$

with 0 ≤ *i, j* ≤ 3 and

(7) $$w_{\text{ang}}(\alpha )=\sum_{r=-1}^{1}w(\frac{8\alpha }{2\pi }+8r)$$

where *x*_*i*_ and *y*_*i*_ are the spatial bin centers located in $x_{i},y_{j}\in \left\{-\frac{3}{2},-\frac{1}{2},\frac{1}{2},\frac{3}{2}\right\}$ and the interpolating function *w*(·) is defined as *w*(*z*) = max(0, 1−|*z*|). The function parameter **v** = (*v*_*x*_, *v*_*y*_) is a vector variable and α a scalar variable. Vector **v** holds pixel coordinates (*v*_*x*_, *v*_*y*_) normalized between −2 and 2 and combined with the function *w*(*z*) it produces zero for every combination of (*i, j*) except for the 4 adjacent spatial bins. On the other hand, *r* is an integer that can vary freely in the set {−1, 0, 1} and α is the difference between the gradient orientation angle and the angle bin center in radians. By following this procedure, summands on Equation (7) are nullified except for the 2 adjacent angular bins.

These binning functions conform the trilinear interpolation that has a combined effect of sharing the contribution of each oriented gradient between their eight adjacent bins in a tridimensional cube in the histogram space, and zero everywhere else (Mortensen and Shapiro, 2005).

The fixed value of 3 is a magnification factor which corresponds to the number of pixels per each block when *s* = 1. As the patch has 16 blocks and 8 bin angles are considered, for each location *l* and channel *c* a feature called *descriptor* **d**^(*l, c*)^ of 128 dimension is obtained. The main differences between this implementation and the standard SIFT Descriptor are described in the Appendix.

**Figure 3:**
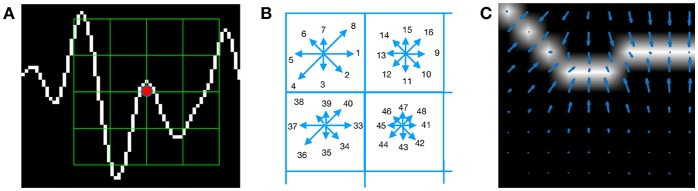
**(A)** Example of a plot of the signal, a keypoint and the corresponding patch. **(B)** A scheme of the orientation's histogram computation. Only the upper-left four blocks are visible. The first eight orientations of the first block, are labeled from 1 to 8 clockwise. The orientation of the second block *B*_1,2_ is labeled from 9 to 16. This labeling continues left-to-right, up-down until the eight orientations for all the sixteen blocks are assigned. They form the corresponding descriptor of 128 coordinates. The length of each arrow represents the value of the histogram on each direction for each block. **(C)** Vector field of oriented gradients. Each pixel is assigned an orientation and magnitude calculated using finite differences.

Figure 3 shows an example of a patch and a scheme of the histogram computation. In Figure 3A a plot of the signal and the patch centered around the keypoint is shown. In Figure 3B the possible orientations on each patch are illustrated. Only the upper-left four blocks are visible. The first eight orientations of the first block, are labeled from 1 to 8 clockwise. The orientations of the second block *B*_1, 2_ are labeled from 9 to 16. This labeling continues left-to-right, up-down until the eight orientations for all the sixteen blocks are assigned. They form the corresponding descriptor **d** of 128 coordinates. Finally, in (C) an enlarged image plot is shown where the oriented gradient vector for each pixel can be seen.

#### 2.1.4. Speller Matrix Letter Identification

##### 2.1.4.1. P300 ERP extraction

Segments corresponding to row flickering are labeled 1–6, whereas those corresponding to column flickering are labeled 7–12. The extraction process has the following steps:

- **Step A:** First highlight rows and columns from the matrix in a random permutation order and obtain the Ensemble Average as detailed in steps 1, 2, and 3 in section 2.1.1.
- **Step B:** Plot the signals ${\tilde{x}}^{l}\left(n,c\right)$, 1 ≤ *n* ≤ *n*_*max*_, 1 ≤ *c* ≤ *C*, according section 2.1.2 in order to generate the images *I*^(*l, c*)^ for rows and columns 1 ≤ *l* ≤ 12.
- **Step C:** Obtain the descriptors **d**^(*l, c*)^ for rows and columns from *I*^(*l, c*)^ in accordance to the method described in section 2.1.3.

##### 2.1.4.2. Calibration

A trial, as defined by the BCI2000 platform (Schalk et al., 2004), is every attempt to select just one letter from the speller. A set of trials is used for calibration and once the calibration is complete it can be used to identify new letters from new trials.

During the calibration phase, two descriptors **d**^(*l,c*)^ are extracted for each available channel, corresponding to the locations *l* of a selection of one previously instructed letter from the set of calibration trials. These descriptors are the P300 templates, grouped together in a template set called *T*^*c*^. The set is constructed using the steps described in section 2.1.1 and the steps A, B, and C of the P300 ERP extraction process.

Additionally, the best performing channel, *bpc* is identified based on the the channel where the best Character Recognition Rate is obtained.

##### 2.1.4.3. Letter identification

In order to identify the selected letter, the template set *T*^*bpc*^ is used as a database. Thus, new unclassified descriptors **q**^(*l,bpc*)^ are computed and they are compared against the descriptors belonging to the calibration template set *T*^*bpc*^.

The Naive Bayes Nearest Neighbor (k-NBNN) (Boiman et al., 2008) is a discriminative (Wolpaw and Wolpaw, 2012) semi-supervised classification algorithm that allows the categorization of an image to one class by comparing the set of extracted descriptors to those which are more similar from template dictionaries. This work proposes an adapted version to obtain a unary classification scheme to identify the selected letter in the P300-Based BCI Speller, based on the features provided by the calculated descriptors.

- **Step D:** Match to the calibration template *T*^*bpc*^ by computing
  (8) $$\hat{row}=\arg\underset{{\;}_{l\in \{1,\ldots ,6\}}}{\min}\sum_{h=1}^{k}{\left\|q^{\left(l,bpc\right)}-d_{h}^{\left(bpc\right)}\right\|}^{2}$$
  and
  (9) $$\hat{col}=\arg\underset{{\;}_{l\in \{7,\ldots ,12\}}}{\min}\sum_{h=1}^{k}{\left\|q^{\left(l,bpc\right)}-d_{h}^{\left(bpc\right)}\right\|}^{2}$$
  with ${\text{d}}_{h}^{\left(bpc\right)}$ belonging to the set $N_{T}\left({\text{q}}^{\left(l,bpc\right)}\right)$, which is defined, for the best performing channel, as $N_{T}(q^{\left(l,bpc\right)})=\{d_{h}^{\left(bpc\right)}\in T^{bpc}/d^{\left(bpc\right)}$ is the *k*-nearest neighbor of **q**^(*l, bpc*)^}. This set is obtained by sorting all the elements in *T*^*bpc*^ based on distances between them and **q**^(*l, bpc*)^, choosing the *k* with smaller values, with *k* a parameter of the algorithm.

By computing the aforementioned equations, the letter of the matrix can be determined from the intersection of the row $\hat{row}$ and column $\hat{col}$. Figure 2 shows a scheme of this process.

### 2.2. Experimental Protocol

To verify the validity of the proposed framework and method, the public dataset 008-2014 (Riccio et al., 2013) published on the BNCI-Horizon website (Brunner et al., 2014) by IRCCS Fondazione Santa Lucia, is used. Additionally, an own dataset with the same experimental conditions is generated. Both of them are utilized to perform an offline BCI Simulation to decode the spelled words from the provided signals.

The algorithm is implemented on MATLAB V2017a (Mathworks Inc., Natick, MA, USA). The algorithm described in section 2.1.3 is implemented on a modified version of the VLFeat (Vedaldi and Fulkerson, 2010) Computer Vision library. Furthermore, in order to enhance the impact of this paper and for a sake of reproducibility, the code of the entire algorithm, including the modified VLFeat library, has been made available at: https://bitbucket.org/itba/hist.

In the following sections the characteristics of the datasets and parameters of the identification algorithm are described.

#### 2.2.1. P300 ALS Public Dataset

The experimental protocol used to generate this dataset is explained in Riccio et al. (2013) but can be summarized as follows: eight subjects with confirmed diagnoses but on different stages of ALS disease, were recruited and accepted to perform the experiments. The Visual P300 detection task designed for this experiment consisted of spelling seven words of five letters each, using the traditional P300 Speller Matrix (Farwell and Donchin, 1988). The flashing of rows and columns provide the deviant stimulus required to elicit this physiological response. The first 3 words are used for calibration and the remaining four words, for testing with visual feedback. A trial is every attempt to select a letter from the speller. It is composed of signal segments corresponding to *k*_*a*_ = 10 repetitions of flashes of 6 rows and *k*_*a*_ = 10 repetitions of flashes of 6 columns of the matrix, yielding 120 repetitions. Flashing of a row or a column is performed for 0.125 s, following by a resting period (i.e., inter-stimulus interval) of the same length. After 120 repetitions an inter-trial pause is included before resuming with the following letter.

The recorded dataset was sampled at 256 Hz and it consisted of a scalp multichannel EEG signal for electrode channels Fz, Cz, Pz, Oz, P3, P4, PO7, and PO8, identified according to the 10–20 International System, for each one of the eight subjects. The recording device was a research-oriented digital EEG device (g.Mobilab, g.Tec, Austria) and the data acquisition and stimuli delivery were handled by the BCI2000 open source software (Schalk et al., 2004).

In order to assess and verify the identification of the P300 response, subjects are instructed to perform a copy-spelling task. They have to fix their attention to successive letters for copying a previously determined set of words, in contrast to a free-running operation of the speller where each user decides on its own what letter to choose.

#### 2.2.2. P300 for Healthy Subjects

We replicate the same experiment on healthy subjects using a wireless digital EEG device (g.Nautilus, g.Tec, Austria). The experimental conditions are the same as those used for the previous dataset, as detailed in section 2.2.1. The produced dataset is available in a public online repository (Ramele et al., 2017).

Participants are recruited voluntarily and the experiment is conducted anonymously in accordance with the Declaration of Helsinki published by the World Health Organization. No monetary compensation is handed out and all participants agree and sign a written informed consent. This study is approved by the *Departamento de Investigación y Doctorado, Instituto Tecnológico de Buenos Aires (ITBA)*. All healthy subjects have normal or corrected-to-normal vision and no history of neurological disorders. The experiment is performed with 8 subjects, 6 males, 2 females, 6 right-handed, 2 left-handed, average age 29.00 years, standard deviation 11.56 years, range 20–56 years.

EEG data is collected in a single recording session. Participants are seated in a comfortable chair, with their vision aligned to a computer screen located one meter in front of them. The handling and processing of the data and stimuli is conducted by the OpenVibe platform (Renard et al., 2010).

Gel-based active electrodes (g.LADYbird, g.Tec, Austria) are used on the same positions Fz, Cz, Pz, Oz, P3,P4, PO7, and PO8. Reference is set to the right ear lobe and ground is preset as the AFz position. Sampling frequency is slightly different, and is set to 250 Hz, which is the closest possible to the one used with the other dataset.

#### 2.2.3. Parameters

The patch size is *X*_*P*_ = 12*s* × 12*s* pixels, where *s* is the scale of the local patch and it is an input parameter of the algorithm. The P300 event can have a span of 400 ms and its amplitude can reach 10μ*V* (Rao, 2013). Hence it is necessary to utilize a signal segment of size *t*_*max*_ = 1 second and a size patch *X*_*P*_ that could capture an entire transient event. With this purpose in consideration, the *s* value election is essential.

**Figure 4:**
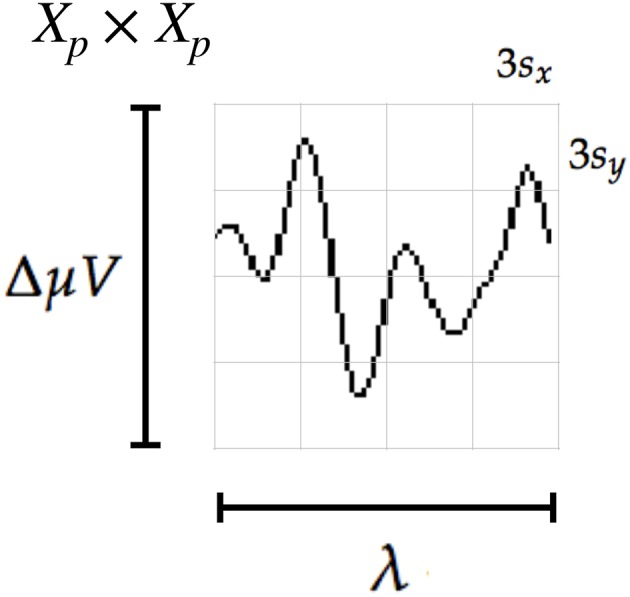
The scale of local patch is selected in order to capture the whole transient event. The size of the patch is *X*_*p*_ × *X*_*p*_ pixels. The vertical size consists of four blocks of size 3*s*_*y*_ pixels which is high enough as to contain the signal Δ*μV*, the peak-to-peak amplitude of the transient event. The horizontal size includes four blocks of 3*s*_*x*_ and covers the entire duration in seconds of the transient signal event, λ.

We propose the Equations (10) and (11) to compute the scale value in horizontal and vertical directions, respectively.

(10) $$\begin{array}{r} s_{x}=\frac{\gamma \lambda \;F_{s}}{12} \end{array}$$

(11) $$\begin{array}{r} s_{y}=\frac{\gamma \Delta \mu V}{12} \end{array}$$

where λ is the length in seconds covered by the patch, *F*_*s*_ is the sampling frequency of the EEG signal (downsampled to 16 Hz) and Δ*μV* corresponds to the amplitude in microvolts that can be covered by the height of the patch. The geometric structure of the patch is determined by the waveform to be captured, thus we discerned that by using *s* = *s*_*x*_ = *s*_*y*_ = 3 and γ = 4, the local patch and the descriptor can identify events of 9 μ*V* of amplitude, with a span of λ = 0.56 s. This also determines that 1 pixel represents $\frac{1}{\gamma }=\frac{1}{4}\mu V$ on the vertical direction and $\frac{1}{F_{s}\;\gamma }=\frac{1}{64}$ s on the horizontal direction. The keypoints **p_*k*_** are located at $\left(x_{p_{k}},y_{p_{k}}\right)=\left(0.55F_{s}\;\gamma ,z^{l}\left(c\right)\right)=\left(35,z^{l}\left(c\right)\right)$ for the corresponding channel *c* and location *l* (see Equation 4). In this way the whole transient event is captured. Figure 4 shows a patch of a signal plot covering the complete amplitude (vertical direction) and the complete span of the signal event (horizontal direction).

The number of channels *C* is equal to 8 for both datasets, and the number of intensification sequences *k*_*a*_ is fixed to 10. The parameter *k* used to construct the set $N_{T}\left({\text{q}}^{\left(l,c\right)}\right)$ is assigned to *k* = 7, which was found empirically to achieve better results. In addition, the norm used on Equations (8) and (9) is the cosine norm, and descriptors are normalized to [−1, 1].

Lastly, in order to assess the validity of the HIST method, the character recognition rate for both datasets is evaluated replicating the methodology proposed by the ALS dataset's publisher, since authors Riccio et al. (2013) did not report the Character Recognition Rate obtained for this dataset. Frequency filtering, data segmentation and artifact rejection is conducted according to section 2.1.1 yielding 16 x 8 samples per epoch. A multichannel feature consists of time points vector (Lotte et al., 2018), formed by concatenating all the channels (Krusienski et al., 2006). A single-channel variant consists of using time points from a single electrode and performing the analysis on a channel-by-channel basis. Three classification schemes are considered as well. A multichannel version of the Stepwise Linear Discriminant Analysis (SWLDA) classification algorithm. SWLDA is the methodology proposed by the ALS dataset's publisher. Additionally, a single-channel and a multichannel variant of a linear kernel Support Vector Machine (SVM) (Scholkopf and Smola, 2001) classifier are utilized. SVM has been successfully used in several BCI Competitions (Rakotomamonjy and Guigue, 2008).

## 3. Results

Table 1 shows the results of applying the HIST algorithm to the subjects of the public dataset of ALS patients. The percentage of correctly spelled letters is calculated while performing an offline BCI Simulation. From the seven words for each subject, the first three are used for calibration, and the remaining four are used for testing. The best performing channel *bpc* is informed as well. The target ratio is 1:36; hence theoretical chance level is 2.8%. It can be observed that the best performance of the letter identification method is reached in a dissimilar channel depending on the subject being studied. Tables 1, 2 show for comparison the obtained performance rates using single-channel signals with the SVM classifier. The best performing channel, where the best letter identification rate was achieved, is also depicted.

**Table 1:** Character recognition rates for the public dataset of ALS patients using the Histogram of Gradient (HIST) calculated from single-channel plots.

**Participant**	***bpc***	**HIST (%)**	***bpc***	**Single channel SVM (%)**
1	Cz	35	Cz	15
2	Fz	85	PO8	25
3	Cz	25	Fz	5
4	PO8	55	Oz	5
5	PO7	40	P3	25
6	PO7	60	PO8	20
7	PO8	80	Fz	30
8	PO7	95	PO7	85

*Performance rates using single-channel signals with the SVM classifier are shown for comparison. The best performing channel bpc for each method is visualized*.

**Table 2:** Character recognition rates for the own dataset of healthy subjects using the Histogram of Gradient (HIST) calculated from single-channel plots.

**Participant**	***bpc***	**HIST (%)**	***bpc***	**Single channel SVM (%)**
1	Oz	40	Cz	10
2	PO7	30	Cz	5
3	P4	40	P3	10
4	P4	45	P4	35
5	P4	60	P3	10
6	Pz	50	P4	25
7	PO7	70	P3	30
8	P4	50	PO7	10

*Performance rates using single-channel signals with the SVM classifier are shown for comparison. The best performing channel bpc for each method is visualized*.

**Figure 5:**
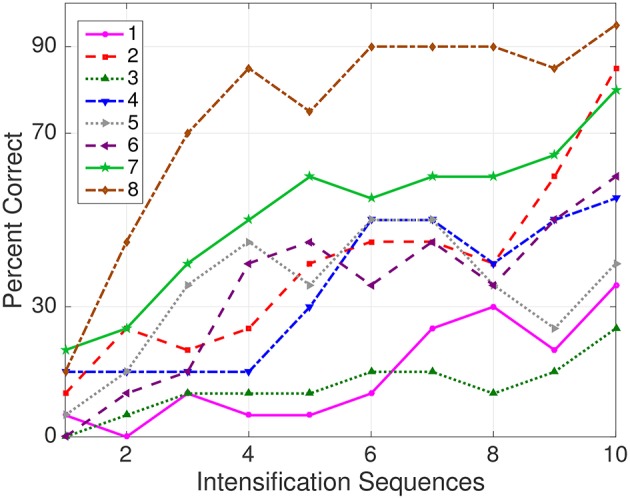
Performance curves for the eight subjects included in the dataset of ALS patients. Three out of eight subjects achieved the necessary performance to implement a valid P300 speller.

The Information Transfer Rate (ITR), or Bit Transfer Rate (BTR), in the case of reactive BCIs (Wolpaw and Wolpaw, 2012) depends on the amount of signal averaging required to transmit a valid and robust selection. Figure 5 shows the performance curves for varying intensification sequences for the subjects included in the dataset of ALS patients. It can be noticed that the percentage of correctly identified letters depends on the number of intensification sequences that are used to obtain the averaged signal. Moreover, when the number of intensification sequences tend to 1, which corresponds to single-intensification character recognition, the performance is reduced. As mentioned before, the SNR of the P300 obtained from only one segment of the intensification sequence is very low and the shape of its P300 component is not very well defined.

In Table 2 the results obtained for 8 healthy subjects are shown. It can be observed that the performance is above chance level. It is verified that HIST method has an improved performance at letter identification than SVM that process the signals on a channel by channel strategy (Wilcoxon signed-rank test, *p* = 0.004 for both datasets).

Tables 3, 4 are presented in order to compare the performance of the HIST method versus multichannel SWLDA and SVM classification algorithms for both datasets. It is verified for the dataset of ALS patients that it has similar performance against other methods like SWLDA or SVM, which use a multichannel feature (Quade test with *p* = 0.55) whereas for the dataset of healthy subjects significant differences are found (Quade test with *p* = 0.02) where only the HIST method achieves a different performance than SVM (with multiple comparisons, significant difference of level 0.05).

**Table 3:** Character recognition rates and the best performing channel *bpc* for the public dataset of ALS patients using the Histogram of Gradient (HIST) (repeated here for comparison purposes).

**Participant**	***bpc* for HIST**	**HIST (%)**	**Multichannel SWLDA (%)**	**Multichannel SVM (%)**
1	Cz	35	45	40
2	Fz	85	30	50
3	Cz	25	65	55
4	PO8	55	40	50
5	PO7	40	35	45
6	PO7	60	35	70
7	PO8	80	60	35
8	PO7	95	90	95

*Performance rates obtained by SWLDA and SVM classification algorithms with a multichannel concatenated feature*.

**Table 4:** Character recognition rates and the best performing channel *bpc* for the own dataset of healthy subjects using the Histogram of Gradient (HIST) (repeated here for comparison purposes).

**Participant**	***bpc* for HIST**	**HIST (%)**	**Multichannel SWLDA (%)**	**Multichannel SVM (%)**
1	Oz	40	65	40
2	PO7	30	15	10
3	P4	40	50	25
4	P4	45	40	20
5	P4	60	30	20
6	Pz	50	35	30
7	PO7	70	25	30
8	P4	50	35	20

*Performance rates obtained by SWLDA and SVM classification algorithms with a multichannel concatenated feature*.

The P300 ERP consists of two overlapping components: the P3a and P3b, the former with frontocentral distribution while the later stronger on centroparietal region (Polich, 2007). Hence, the standard practice is to find the stronger response on the central channel Cz (Riccio et al., 2013). However, Krusienski et al. (2006) show that the response may also arise in occipital regions. We found that by analyzing only the waveforms, occipital channels PO8 and PO7 show higher performances for some subjects.

**Figure 6:**
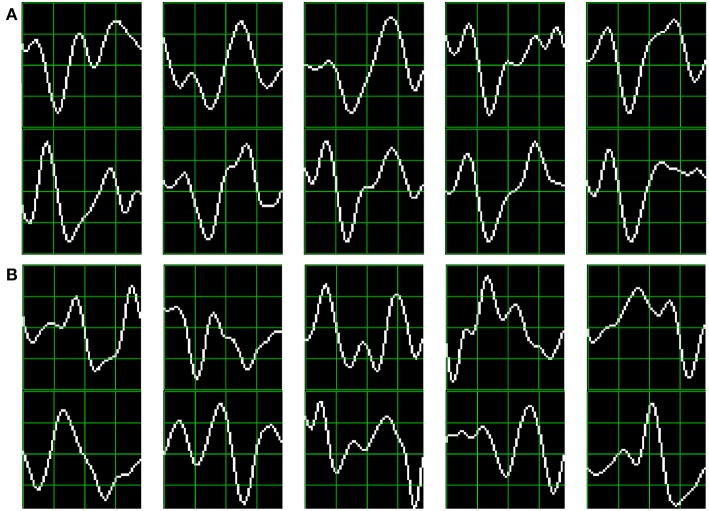
Ten sample P300 template patches for subjects 8 **(A)** and 3 **(B)** of the ALS Dataset. Downward deflection is positive polarity.

As subjects have varying *latencies* and *amplitudes* of their P300 components, they also have a varying stability of the *shape* of the generated ERP (Nam et al., 2010). Figure 6 shows 10 sample P300 templates patches for patients 8 and 3 from the dataset of ALS patients. It can be discerned that in coincidence with the performance results, the P300 signature is more clear and consistent for subject 8 (A) while for subject 3 (B) the characteristic pattern is more difficult to perceive.

Additionally, the stability of the P300 component waveform has been extensively studied in patients with ALS (Sellers et al., 2006; Madarame et al., 2008; Nijboer and Broermann, 2009; Mak et al., 2012; McCane et al., 2015) where it was found that these patients have a stable P300 component, which were also sustained across different sessions. In line with these results we do not find evidence of a difference in terms of the performance obtained by analyzing the waveforms (HIST) for the group of patients with ALS and the healthy group of volunteers (Mann–Whitney *U*-Test, *p* = 0.46). Particularly, the best performance is obtained for a subject from the ALS dataset for which, based on visual observation, the shape of they P300 component is consistently identified.

It is important to remark that when applied to binary images obtained from signal plots, the feature extraction method described in section 2.1.3 generates sparse descriptors. Under this subspace we found that using the cosine metric yielded a significant performance improvement. On the other hand, the unary classification scheme based on the NBNN algorithm proved very beneficial for the P300 Speller Matrix. This is due to the fact that this approach solves the unbalance dataset problem which is inherent to the oddball paradigm (Tibon and Levy, 2015).

## 4. Discussion

Among other applications of Brain Computer Interfaces, the goal of the discipline is to provide communication assistance to people affected by neuro-degenerative diseases, who are the most likely population to benefit from BCI systems and EEG processing and analysis.

In this work, a method to extract an objective metric from the waveform of the plots of EEG signals is presented. Its usage to implement a valid P300-Based BCI Speller application is expounded. Additionally, its validity is evaluated using a public dataset of ALS patients and an own dataset of healthy subjects.

It was verified that this method has an improved performance at letter identification than other methods that process the signals on a channel by channel strategy, and it even has a comparable performance against other methods like SWLDA or SVM, which uses a multichannel feature. Furthermore, this method has the advantage that shapes of waveforms can be analyzed in an objective way. We observed that the shape of the P300 component is more stable in occipital channels, where the performance for identifying letters is higher. We additionally verified that ALS P300 signatures are stable in comparison to those of healthy subjects.

We believe that the use of descriptors based on histogram of gradient orientation, presented in this work, can also be utilized for deriving a shape metric in the space of the P300 signals which can complement other metrics based on time-domain as those defined by Mak et al. (2012). It is important to notice that the analysis of waveform shapes is usually performed in a qualitative approach based on visual inspection (Sellers et al., 2006), and a complementary methodology which offer a quantitative metric will be beneficial to these routinely analysis of the waveform of ERPs.

The goal of this work is to answer the question if a P300 component could be solely determined by inspecting automatically their waveforms. We conclude affirmatively, though two very important issues still remain:

First, the stability of the P300 in terms of its shape is crucial: the averaging procedure, montages, the signal to noise ratio and spatial filters all of them are non-physiological factors that affect the stability of the shape of the P300 ERP. We tested a preliminary approach to assess if the morphological shape of the P300 of the averaged signal can be stabilized by applying different alignments of the stacked segments (see Figure 2) and we verified that there is a better performance when a correct segment alignment is applied. We applied Dynamic Time Warping (DTW) (Casarotto et al., 2005) to automate the alignment procedure but we were unable to find a substantial improvement. Further work to study the stability of the shape of the P300 signature component needs to be addressed.

The second problem is the amplitude variation of the P300. We propose a solution by standardizing the signal, shown in Equation (2). It has the effect of normalizing the peak-to-peak amplitude, moderating its variation. It has also the advantage of reducing noise that was not reduced by the averaging procedure. It is important to remark that the averaged signal variance depends on the number of segments used to compute it (Van Drongelen, 2006). The standardizing process converts the signal to unit signal variance which makes it independent of the number *k*_*a*_ of signals averaged. Although this is initially an advantageous approach, the standardizing process reduces the amplitude of any significant P300 complex diminishing its automatic interpretation capability.

To further extend the capabilities of this method, it would be desirable to implement a multichannel version. The straightforward extension of concatenating the obtained descriptors results in high dimensional feature vector, while other variants that merge descriptors per channel may diminish the mutual information between different channels. Hitherto variants using color versions of SIFT (Van De Sande et al., 2010), where different color bands are mapped to electrode channels, have been explored without substantial success.

In our opinion, the best benefit of the presented method is that a closer collaboration of the field of BCI with physicians can be fostered (Chavarriaga et al., 2017), since this procedure intent to imitate human visual observation. Automatic classification of patterns in EEG that are specifically identified by their shapes like K-Complex, Vertex Waves, Positive Occipital Sharp Transient (Hartman, 2005) are a prospect future work to be considered. We are currently working in unpublished material analyzing K-Complex components that could eventually provide assistance to physicians to locate these EEG patterns, specially in long recording periods, frequent in sleep research (Michel and Murray, 2012). Additionally, it can be used for artifact removal which is performed on many occasions by visually inspecting signals. This is due to the fact that the descriptors are a direct representation of the shape of signal waveforms. In line with these applications, it can be used to build a database (Chavarriaga et al., 2017) of quantitative representations of waveforms and improve atlases (Hartman, 2005), which are currently based on qualitative descriptions of signal shapes.

## Ethics Statement

Participants are recruited voluntarily and the experiment is conducted anonymously in accordance with the declaration of Helsinki published by the World Health Organization. No monetary compensation is handed out and all participants agree and sign a written informed consent. This study is approved by the Departamento de Investigación y Doctorado, Instituto Tecnológico de Buenos Aires (ITBA).

## Author Contributions

This work was part of the Ph.D. thesis of RR which is directed by JS and co-directed by AV.

### Conflict of Interest Statement

The authors declare that the research was conducted in the absence of any commercial or financial relationships that could be construed as a potential conflict of interest.

## Appendix

This section describes the differences between the HIST algorithm proposed in this work and the SIFT Descriptor (Vedaldi and Fulkerson, 2010).

The two most important modifications are:

- SIFT Detector and custom frame: The SIFT Detector provides the keypoint localization information in the standard SIFT method. The keypoint localization information is stored in a *frame* data structure which is composed of the keypoint center location (*x*_*kp*_, *y*_*kp*_), patch scale *s* and patch orientation ϕ: (*x*_*kp*_, *y*_*kp*_, *s*, ϕ). In the HIST proposal the keypoint location and patch parameters are directly specified over the plot image in order to detect the signal waveform (see section 2.2.3) and the SIFT Detector is not used.
- Patch scale: Whereas in the standard SIFT implementation the patch is a squared region and there is only one SIFT scale parameter, in HIST a different scale parameter can be assigned to the horizontal and vertical axis. This is a very important modification because otherwise signal plots which extend only on the horizontal direction of the plot image could not be entirely covered. By using a rectangular patch, there isn't any constraint on its size and it can be adjusted by neurophysiological priors to map any expected waveform based on equations (10) and (11).

Additionally, other changes are implemented where specific steps of the SIFT Descriptor were not found to be useful to characterize signal waveforms:

- Patch orientation: We verified experimentally that the patch orientation ϕ does not provide any extra utility for the extraction of characteristic waveforms from plots. Hence, this patch orientation is fixed to zero (vertical, pointing upwards in Figure 4).
- Rotations: SIFT was designed to allow affine invariance, i.e., to be robust to rotations and scale modifications of patterns in images. It was not found, so far, of any utility to rotate the patch to capture the signal waveform.
- Octave selection: A gradient image is used to obtain the oriented gradients and calculate the histogram of gradient orientations. In SIFT, these gradient images are downsampled and smoothed by a Gaussian filter. The SIFT Descriptor calls *octave* to each downsampling level (Lowe, 2004; Rey-Otero and Delbracio, 2014). The standard SIFT Descriptor estimates the octave to use on the gradient image based on the image size and patch parameters. The HIST method uses only the zero octave which means that the gradient image has the same size as the original image, without any downsampling operation.
- Gradient image smoothing: Additionally, the SIFT Descriptor performs an initial smoothing operation by applying a Gaussian filter on the gradient image regardless of the octave. In the HIST method, this operation is not implemented.
- Descriptor Gaussian weighting: On the standard SIFT Descriptor, a Gaussian weighting operation is performed on the calculated SIFT descriptor to increase the importance of gradients from pixels closer to the center of the patch. For the HIST method, this is found to be in detriment of the waveform characterization and is not used.
- SIFT descriptor codification: The SIFT descriptor *d* is a 128-dimension feature vector, as described in section 2.1.3. Histogram values are floating point numbers, all positive, and they are accumulated on each coordinate of this vector. Once all the gradients are summarized, the following operations are performed:
  - The descriptor is ℓ-2 normalized (i.e., all the values are divided by the euclidean norm of the descriptor).
  - Each value is clamped to 0.2. This means that any value above 0.2 is set to 0.2.
  - The descriptor is ℓ-2 re-normalized again (Rey-Otero and Delbracio, 2014).

This generates a 128-vector of floating point numbers, between [0, 1]. In the HIST implementation, these values are rescaled to [−1, 1] in order to use the cosine distance (Arandjelovic and Zisserman, 2012) on Equations (9) and (8). Finally, output values are cast to floating point numbers (i.e., floats). yielding an effective 128-vector of floats between [−1, 1].
